# Texture-defined objects influence responses of blowfly motion-sensitive neurons under natural dynamical conditions

**DOI:** 10.3389/fnint.2014.00034

**Published:** 2014-04-29

**Authors:** Thomas W. Ullrich, Roland Kern, Martin Egelhaaf

**Affiliations:** Department of Neurobiology and Center of Excellence Cognitive Interaction Technology, Bielefeld UniversityBielefeld, Germany

**Keywords:** blowfly, contrast, neural activity, optic flow, spatial discontinuity, spatial frequency, vision

## Abstract

The responses of visual interneurons of flies involved in the processing of motion information do not only depend on the velocity, but also on other stimulus parameters, such as the contrast and the spatial frequency content of the stimulus pattern. These dependencies have been known for long, but it is still an open question how they affect the neurons’ performance in extracting information about the structure of the environment under the specific dynamical conditions of natural flight. Free-flight of blowflies is characterized by sequences of phases of translational movements lasting for just 30–100 ms interspersed with even shorter and extremely rapid saccade-like rotational shifts in flight and gaze direction. Previous studies already analyzed how nearby objects, leading to relative motion on the retina with respect to a more distant background, influenced the response of a class of fly motion sensitive visual interneurons, the horizontal system (HS) cells. In the present study, we focused on objects that differed from their background by discontinuities either in their brightness contrast or in their spatial frequency content. We found strong object-induced effects on the membrane potential even during the short intersaccadic intervals, if the background contrast was small and the object contrast sufficiently high. The object evoked similar response increments provided that it contained higher spatial frequencies than the background, but not under reversed conditions. This asymmetry in the response behavior is partly a consequence of the depolarization level induced by the background. Thus, our results suggest that, under the specific dynamical conditions of natural flight, i.e., on a very short timescale, the responses of HS cells represent object information depending on the polarity of the difference between object and background contrast and spatial frequency content.

## INTRODUCTION

Gaining reliable information about the environment is crucial for all flying animals, such as flies, because they need to behave in an adaptive way in a wide range of environments, for instance, when searching for suitable landing sites or when attempting to avoid collisions with obstacles. The image displacements on the eyes induced by self-motion of the animal (“optic flow”) are a rich source of information, especially if spatial tasks have to be solved in fast flight. Optic flow does not only provide information about the animal’s self-motion, but also about the spatial layout of the surrounding world. While the translational self-motion components are leading to optic flow that contains spatial information, the optic flow components induced by rotational motion are independent from the spatial structure (Koenderink, 1986). This basic geometrical fact gains particular relevance for the specific mode of self-motion of many insects: flies, but also bees, exhibit a saccadic flight style, with brief flight phases of strong rotations (“saccades”) alternating with intersaccadic intervals of almost pure translational motion (Land, 1973; Schilstra and Van Hateren, 1999; Van Hateren and Schilstra, 1999; Tammero and Dickinson, 2002; Mronz and Lehmann, 2008; Boeddeker et al., 2010; Braun et al., 2010, 2012; Geurten et al., 2010; Kern et al., 2012; van Breugel and Dickinson, 2012). This flight style has been interpreted as a behavioral strategy to separate the translational and rotational optic flow components and, thus, to facilitate processing of spatial information by the nervous system. However, the timescale on which environmental information needs to be extracted from the translational intersaccadic optic flow is short and lasts, on average, for only 30–100 ms (Boeddeker et al., 2005; Kern et al., 2005, 2006, 2012; Karmeier et al., 2006; Egelhaaf et al., 2012; Liang et al., 2012).

The posterior part of the third visual neuropile, the lobula plate, is an important site of motion information processing in the visual system of the fly. At this location, a population of well-characterized cells resides, the lobula plate tangential cells (LPTCs). Within their large receptive fields LPTCs respond directionally selective to visual motion, for example as perceived during self-motion of the animal (Hausen, 1984; Krapp, 2000; Egelhaaf et al., 2002; Egelhaaf, 2006; Borst et al., 2010). As LPTCs respond to both rotational and translational optic flow (Hausen, 1982b; Krapp, 2000; Karmeier et al., 2003), the functional role of these cells is still under debate (Egelhaaf et al., 2012). Traditionally, LPTCs are thought to mainly play a role in extracting information about rotational self-motion of the animal (Krapp and Hengstenberg, 1996; Krapp et al., 1998, 2001). In contrast, LPTCs have also been shown to strongly respond to translational optic flow (Karmeier et al., 2003, 2006; Kern et al., 2005). Since the responses of LPTCs rise with increasing velocity within a certain range, they indirectly convey information about the distance to objects in the environment during the short time intervals of intersaccadic translational self-motion (Kern et al., 2005; van Hateren et al., 2005; Karmeier et al., 2006; Egelhaaf et al., 2012; Liang et al., 2012). Accordingly, when stimulated with optic flow as experienced during free flight, response increments in LPTCs were induced when a nearby object was crossing their receptive field during an intersaccadic interval (Liang et al., 2008, 2012). In these studies, objects were mainly defined by discontinuities in depth which resulted in velocity changes within the optic flow field. However, LPTC responses do not only depend on velocity and, thus, on the distance of objects during translational movements. They are also affected by other image features, such as the brightness contrast and spatial frequency content of the stimulus pattern (Hausen, 1982b; Egelhaaf and Borst, 1989; Hausen and Egelhaaf, 1989; Dror et al., 2001; Meyer et al., 2011; O’Carroll et al., 2011). Hence, objects defined by textural features may also have some impact on intersaccadic LPTC responses. Textural cues, even those of natural sceneries, may lead to pronounced modulations in the time-dependent response profile of LPTCs when stimulated with constant velocity motion for several hundreds of milliseconds or even seconds (Egelhaaf et al., 1989; Single and Borst, 1998; Meyer et al., 2011; O’Carroll et al., 2011). However, this issue has never been addressed so far under the dynamical conditions of natural flight where the intersaccadic time intervals for gathering environmental information do rarely last for more than 100 ms. This will be the main objective of the present study.

Our study systematically addresses how the responses of LPTCs are affected by objects that are defined by brightness contrast or by their spatial frequency characteristics under the dynamical conditions of natural retinal image flow. Although under natural conditions objects may jointly be defined by a variety of visual cues, we excluded differences in depth to focus on the specific texture cues. Therefore, our objects were defined as areas on the wall of a virtual flight arena. They were covered with textures different from the background. As in previous studies on the encoding of environmental information during intersaccadic intervals (Liang et al., 2008, 2011, 2012), we made intracellular electrophysiological recordings from horizontal system (HS) cells, a well-known class of LPTCs (Hausen, 1982a,b; Krapp et al., 2001). These cells are strongly activated by forward translational motion which is the most prominent motion component during intersaccadic intervals (Kern et al., 2005; Karmeier et al., 2006). Moreover, HS cells function as output neurons of the visual system and connect directly to neurons descending to the motor control centers (Haag et al., 2010). We will show in the present study that during the short intersaccadic intervals of natural flights object-induced response increments during intersaccadic intervals are only found on the condition that the background textures did not evoke very large responses on their own. Hence, in a critical way it depends on the combination of background and object texture whether or not an object defined by texture affects the response of the cell.

## MATERIALS AND METHODS

### STIMULATION

**FIGURE 1:**
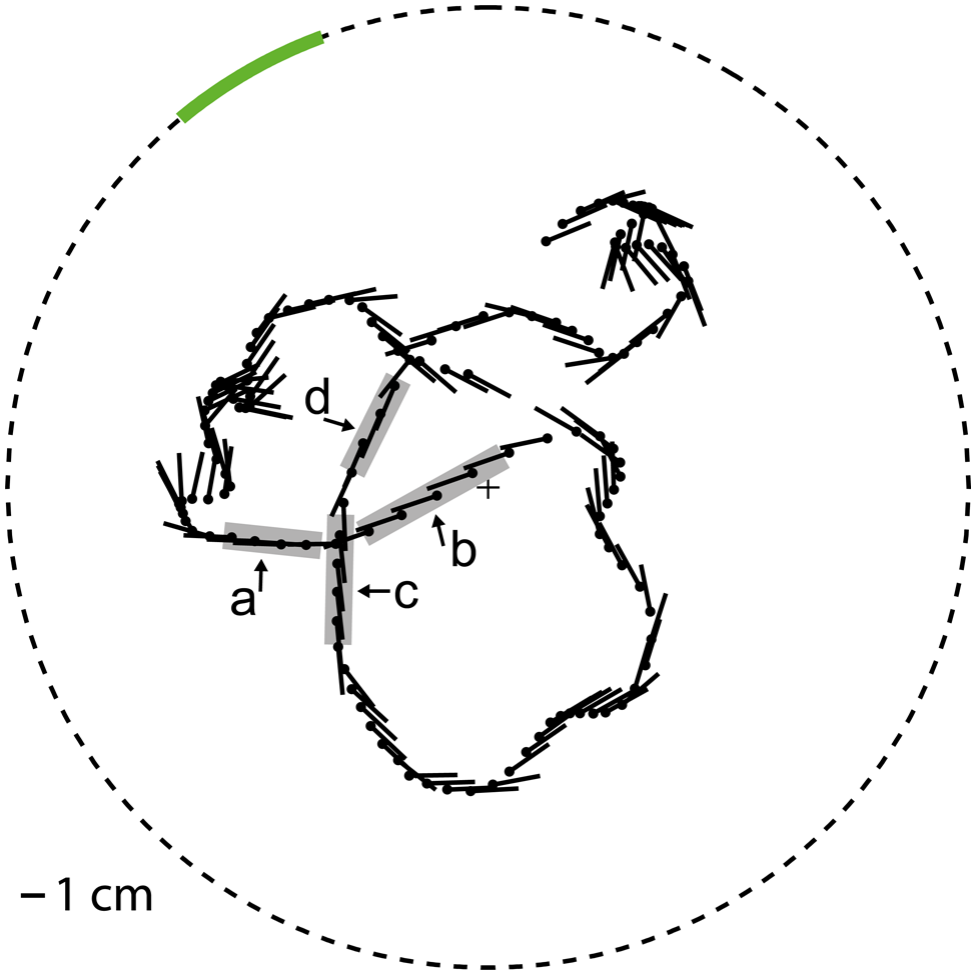
**Virtual flight arena The object area (green line) is embedded in the cylindrical wall (dashed circle).** The position (black dot) and yaw orientation (black line, pointing to the abdomen) of (radius 0.2 m), top view. the fly head is plotted every 25 ms on the flight trajectory. The object response intervals (gray, intervals a–d) are marked at the trajectory.

Movies were generated for motion stimulation that approximated what a blowfly would have seen while moving on a flight trajectory with natural dynamics in a flight arena with experimenter-defined wall textures. The flight trajectory and the corresponding gaze direction of the fly were chosen from a dataset that was recorded in a cubic arena (edge length 0.4 m) in the laboratory of Van Hateren and Schilstra (1999) and already used in previous studies in our lab. This trajectory was centered in a virtual model of a flight arena that was generated for our experiments and differed from the arena the trajectory was recorded in. The virtual setup model for stimulus generation was a cylindrical arena with a radius of 0.20 m and a height of about 1.26 m in which the trajectory was centered (**Figure 1**). The wall of the cylindrical arena was covered with different textures according to the experimental conditions. Both the ceiling and the floor were black. At one azimuthal position on the wall a vertical stripe was defined as the object area covering the whole height of the arena and 20° in horizontal extent when looking from the center of the arena. The remainder of the wall will be referred to as background area. We selected the position of the object area in such a way that its projection on the retina was moving through the receptive field of HS cells in the preferred direction during several intersaccadic intervals. To analyze whether the object affects the cell response we compared the cell signal between two different conditions: Under the “object condition” the texture in the object area differed from the background texture according to our experimental design (see below). In contrast, under the “no-object condition” the textures of the object area and the background area were the same. We used three different types of stimuli.

**FIGURE 2:**
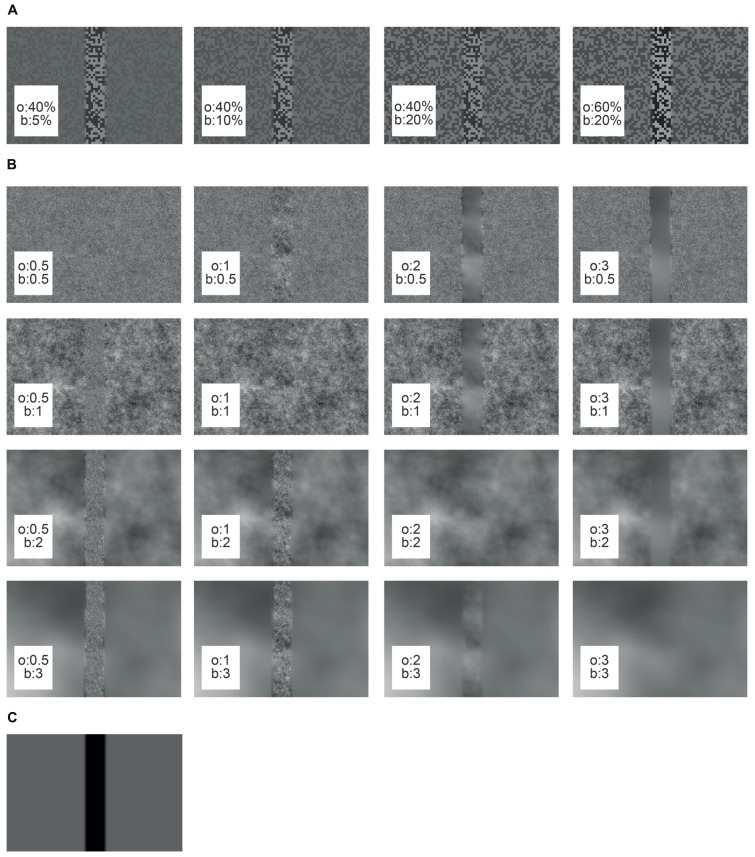
**Cut-outs of different arena wall textures used in this study.** The object texture (o) is embedded in the background texture (b). **(A)** Contrast textures, **(B)** spatial frequency textures, **(C)** reference texture.

Under the “object condition” the object texture differed from the background texture either in its brightness contrast or its spatial frequency composition. The *basic textures for the contrast stimuli* were generated by using a random checkerboard pattern of two brightness values. The RMS contrasts of the bright and the dark squares were set to a variety of values (5, 10, 20, 40, 50, or 60%), while maintaining the mean brightness of the pattern at 4,000 cd/m^2^ (**Figure 2A**). RMS contrast was calculated as the standard deviation of the pixel brightness divided by the average brightness. Different pairs of object and background contrasts were tested (see results section). For reducing brightness discontinuities at the borders of the object area the edges were sigmoidally smoothed across a symmetrical area of 3° to the left and right of the object borders.

The *basic spatial frequency texture* was created from a random noise pattern with a Gaussian distribution of the brightness values of its pixels. The noise pattern was linearly filtered with different characteristics to approximate the power spectra of natural images. The latter can be approximated by the function 1/f^α^, with *f* corresponding to the spatial frequency and α being an exponent that was shown to widely vary around a value of 2 (Ruderman, 1994; van der Schaaf and van Hateren, 1996). The parameter α was set to 0.5, 1, 2, or 3 (**Figure 2B**). After filtering, the pixel values were scaled to maintain the mean brightness level. The edge between object and background area covered by different spatial frequency patterns was smoothed as described above for the contrast stimuli. It should be noted that the edges of the object inevitably added high spatial frequencies to the texture on the condition that object and background textures differed.

For the *reference stimulus* the background area was homogeneous at a mean brightness level, whereas the object area was set to the lowest brightness value (**Figure 2C**). Since under these conditions the edges of the object were the only moving parts of the entire scenery, we used the reference stimulus to infer the time intervals in which object motion was activating the cell and, thus, to select our intersaccadic intervals of interest (see below).

The cell signals induced by our stimuli strongly varied over time due to the dynamics of the natural trajectory they are based on. Thus, we monitored signal quality by presenting a simple *characterizing stimulus* on a regular basis during the course of the experiment. This stimulus was a vertical sinusoidal grating that moved in horizontal direction (wavelength 20°, temporal frequency 1.92 Hz, Michelson contrast 0.98). The pattern rotated in the cell’s preferred as well as in its anti-preferred direction, followed by front-to-back motion on both sides of the head. Recording was stopped in case the maximum depolarization induced by this stimulus differed more than 50% in amplitude from the previous presentation.

To reduce transient brightness changes between consecutive stimulus presentations, a static image with mean brightness of the first frame of the stimulus movie was shown for 1 s followed by a fade-in of the first image for 500 ms. Each stimulus movie lasted for 3.66 s. For technical reasons the stimulus was played approximately 4.5% slower than the corresponding retinal movements as experienced by the fly on the original trajectory. A stationary pattern was presented for 7 s between subsequent stimulus presentations to exclude accumulation of motion adaptation effects. The stimulus movies were presented in pseudorandom order. The characterizing stimulus was shown after having presented each stimulus twice.

The stimulus movies were presented with a custom-made stimulation device called FliMax (Lindemann et al., 2003). This device allows stimulation of extended parts of the visual field of the fly. We used the latest version of FliMax which is equipped with ultra-bright LEDs (maximal luminance ca. 12,000 cd/m^2^, see Liang et al., 2011) and works with an image update rate of 354 Hz. Due to the limited number of LEDs constituting the FliMax stimulator and the angular distance of LEDs of approximately 2° the stimulus textures get low-pass filtered. This filtering inevitably reduces the power of high spatial frequencies which effectively increases the parameter α of the spatial frequency textures. However, since the spatial resolution of FliMax roughly matches the resolution of the eyes of our experimental animal, our conclusions can be assumed not to be affected by this filtering in any qualitative way.

### ELECTROPHYSIOLOGY

Female blowflies of the species *Calliphora vicina* (Robineau-Desvoidy), younger than 48 h, were obtained from our breeding stock. The flies were anesthetized with carbon dioxide and immobilized with beeswax. Legs, proboscis, and antennae were removed to reduce movements of the brain tissue. The head was pulled forward and fixed to the thorax to get free access to the back of the head. The head capsule was opened and the esophagus and some tracheae were removed to get access to the lobula plate. The fly was attached to a holder, such that its head was aligned with reference to the symmetry of the deep pseudopupil (Kirschfeld and Franceschini, 1969). Ringer solution (Kurtz et al., 2000) was used to prevent the tissue from running dry. Electrical responses were recorded intracellularly from HS-cell axons in the right optic lobe of the fly using sharp glass electrodes (GC100TF-10; Clark Electromedical Instruments, Pangbourne Reading, UK). The glass electrodes were filled with 1 M KCl solution and had input resistances between 15 and 42 MΩ. The cells were characterized by their specific response profile: HS cells are activated by ipsilateral wide-field front-to-back (preferred direction) motion and show graded membrane potential depolarizations with superimposed spikes of variable amplitude. Under motion stimulation in the opposite, i.e., the anti-preferred direction, HS cells show hyperpolarizing membrane potential shifts (Hausen, 1982a). Three distinguishable HS cells reside in each hemisphere of the fly brain. Their receptive fields are located in the ventral (HSS), equatorial (HSE), and the dorsal (HSN) part of the visual field (Hausen, 1982b; Krapp et al., 2001). Since under the stimulus conditions of our study all types of HS cells showed similar object-induced responses, the data were pooled across HS cell types. Traces with rapid shifts of the resting potential or strong drifts of the cell depolarization were excluded (see above). Only the recordings of those cells were used for analysis that lasted for more than six repetitions of each stimulus. The recorded response traces were sampled at 8,192 kHz. The temperatures measured near the thorax of the animal during the experiments ranged between 23 and 34°C. The number of the stimulus movies varied between phases of the experiment according to the tested combinations of contrast or spatial frequency textures and the reference stimulus. The exact numbers of the included recordings for each stimulus are given in the result section (**Table 1**).

**Table 1:** Results of two-sided *t*-tests for ΔV ≠ 0, asterisk marks significance (*p* <0.05), number *N* of recorded cells included in calculations and average object induced absolute difference in membrane depolarization without normalization (abs. ΔV) for all combinations of background (bg.) and object (obj.).

bg. contrast = 5%	obj. contrast = 40%	*p* = 0.014*	*N* = 12	abs. ΔV = 2.17 mV
bg. contrast = 10%	obj. contrast = 40%	*p* = 0.661	*N* = 6	abs. ΔV = 0.51 mV
bg. contrast = 20%	obj. contrast = 40%	*p* = 0.139	*N* = 20	abs. ΔV = 0.35 mV
bg. contrast = 20%	obj. contrast = 60%	*p* = 0.027*	*N* = 9	abs. ΔV = 0.75 mV
bg. α = 0.5	obj. α = 1	*p* = 0.733	*N* = 7	abs. ΔV = 0.05 mV
	obj. α = 2	*p* = 0.937	*N* = 7	abs. ΔV = 0.03 mV
	obj. α = 3	*p* = 0.554	*N* = 7	abs. ΔV = 0.23 mV
bg. α = 1	obj. α = 0.5	*p* = 0.761	*N* = 7	abs. ΔV = -0.07 mV
	obj. α = 2	*p* = 0.304	*N* = 11	abs. ΔV = -0.17 mV
	obj. α = 3	*p* = 0.107	*N* = 11	abs. ΔV = -0.50 mV
bg. α = 2	obj. α = 0.5	*p* = 0.103	*N* = 7	abs. ΔV = 0.84 mV
	obj. α = 1	*p* = 0.009*	*N* = 11	abs. ΔV = 0.73 mV
	obj. α = 3	*p* = 0.537	*N* = 11	abs. ΔV = 0.14 mV
bg. α = 3	obj. α = 0.5	*p* <0.001*	*N* = 7	abs. ΔV = 4.04 mV
	obj. α = 1	*p* <0.001*	*N* = 11	abs. ΔV = 4.44 mV
	obj. α = 2	*p* <0.001*	*N* = 11	abs. ΔV = 3.67 mV

### DATA ANALYSIS

#### The intersaccadic response of the cell

**FIGURE 3:**
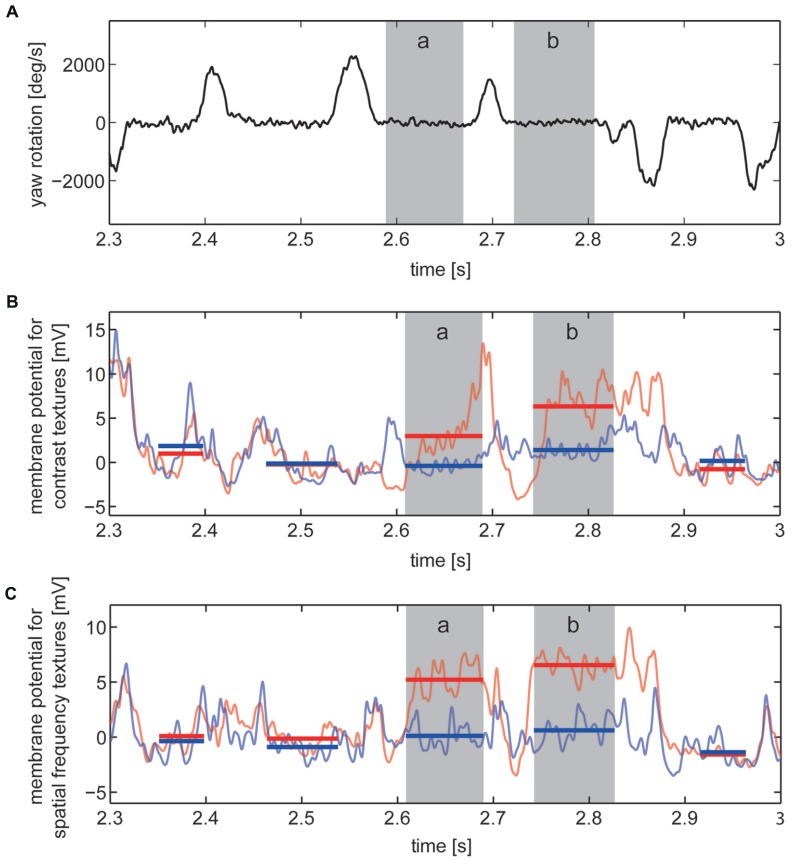
**Responses to section of the image sequences experienced on flight trajectory under contrast stimulus and spatial frequency stimulus conditions.** Two of the selected “object response intervals” are marked (gray, intervals a and b). **(A)** Detail showing the velocity of yaw rotations of the fly with saccadic turns and intersaccadic intervals. **(B,C)** Corresponding cell responses averaged across the recordings obtained from one example cell to stimuli of “no-object condition” (light blue) and “object condition” (light red). Intersaccadic responses to “no-object condition” (blue) and “object condition” (red) marked by horizontal lines covering the periods of every intersaccadic interval within this section of the cell response. **(B)** Cell responses to contrast defined stimuli and **(C)** to stimuli defined by spatial frequency content.

For identifying the intersaccadic intervals in the flight trace the time-dependent yaw velocity was smoothed by averaging across a sliding window with a width of 18 ms and then compared with a threshold of 200°/s. Intervals with yaw velocities exceeding 200°/s were defined as saccades, the other periods as intersaccadic intervals (<200°/s; **Figure 3A**). We ensured that the detection of the intersaccadic intervals did not strongly depend on the exact value of this threshold. The time windows for analyzing the intersaccadic responses were further truncated by cutting 10 ms at their beginning and their end to exclude potential influences of saccades on the intersaccadic signal.

Our basic interest was to assess whether objects exclusively differing from their background by texture parameters, such as contrast or spatial frequency content, were influencing the signal of the cell during intersaccadic intervals. Similar to previous studies (Liang et al., 2008, 2011, 2012), we calculated the object-induced response increment of the cell signal in the following way: The resting potential was calculated by time-averaging the cell response across a time window of 500 ms prior to motion onset of each stimulus repetition, while the stimulus screen was being lit at half maximal brightness. Intersaccadic responses were determined by subtracting the resting potential from the recorded cell response and then by time-averaging within the time windows for the analysis of the intersaccadic responses (**Figures 3B,C**).

#### Selection of the intersaccadic intervals with object responses

**FIGURE 4:**
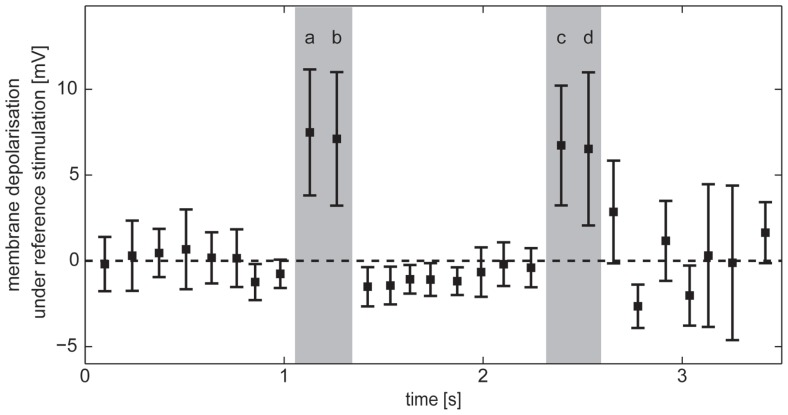
**Horizontal system response in intersaccadic intervals recorded during stimulation with the reference stimulus.** Average responses during individual intersaccadic intervals with the standard deviation across the recordings of HS cells (*n* = 23). Selected “object response intervals” are marked gray.

During self-motion the retinal motion patterns change in a complex fashion, depending on the direction and velocity of self-motion and the distance between arena wall and fly as well as on the wall’s textural properties like the brightness contrast and spatial frequency composition. As a consequence, the responses of HS cells show a complex time structure, and it is not immediately obvious which response components are evoked by an object or by the background. To identify the intersaccadic intervals in which the object affects the response of HS cells, we used the reference stimulus which consisted only of the object while the background was homogeneous and did not lead to motion responses on its own. Thus, the reference stimulus depolarized the cells only if the edges of the object were moving in preferred direction through their receptive field. In this way intersaccadic intervals with object influence on the neural response could be pinpointed. These intervals will be termed “object response intervals.” Since the depolarization amplitude differed between recordings, we normalized the resulting average intersaccadic response to the responses of the cell to preferred direction motion of the characterizing stimulus after subtracting the resting potential (see above). Since the membrane potential also showed spontaneous fluctuations during intersaccadic intervals and since the object might have influenced the cell marginally in some intersaccadic intervals, for instance, if just touching the low-sensitivity borders of the cell’s receptive field, we selected those intervals during which object responses were unambiguous. Four “object response intervals” were selected by the criterion that the average intersaccadic response exceeded the mean response of all average intersaccadic responses by more than the standard deviation across all intersaccadic intervals (**Figure 4**).

#### Determination of the object-induced membrane potential changes

We quantified the effect of the object on the response of the cell by determining the object-induced difference of the membrane potential (ΔV) as follows: We calculated the intersaccadic response difference by subtracting the intersaccadic responses to the “no-object condition” from the intersaccadic responses to the “object condition.” This was done separately for all recordings before averaging. The resulting difference curves were averaged across the recordings from the same cell and are presented as percentage values of the average intersaccadic response to preferred direction motion of the characterizing stimulus after subtracting the resting potential. In this way object-induced responses could be compared between cells. Thus, ΔV is the normalized intersaccadic response difference for the “object condition” and “no-object condition” during the “object response intervals” averaged across the intervals and the different cells in percent of the induced depolarization during stimulation with the characterizing stimulus. The average *absolute* object-induced differences in membrane depolarization (abs. ΔV) were calculated in the same way as ΔV, except omitting the normalization to the characterizing stimulus and are given for all texture combinations in **Table 1**.

All programs for data analysis are based on Matlab (Version 7.13.0.564, The MathWorks). For statistical tests the Matlab statistics toolbox was used.

## RESULTS

Forward motion of the fly leads to front-to-back-motion of the retinal image that depolarized the HS cells strongly. The appearance of objects, defined by changes of certain texture properties in the visual field, induced changes in the retinal input and, thus, affected the response of these cells. We compared the influence of different objects defined by changes in the brightness contrast and the spatial frequency composition on the cell response with ΔV quantifying the object-induced difference of the average cell depolarization in object response intervals.

### RESPONSES TO OBJECTS DEFINED BY BRIGHTNESS CONTRAST

**FIGURE 5:**
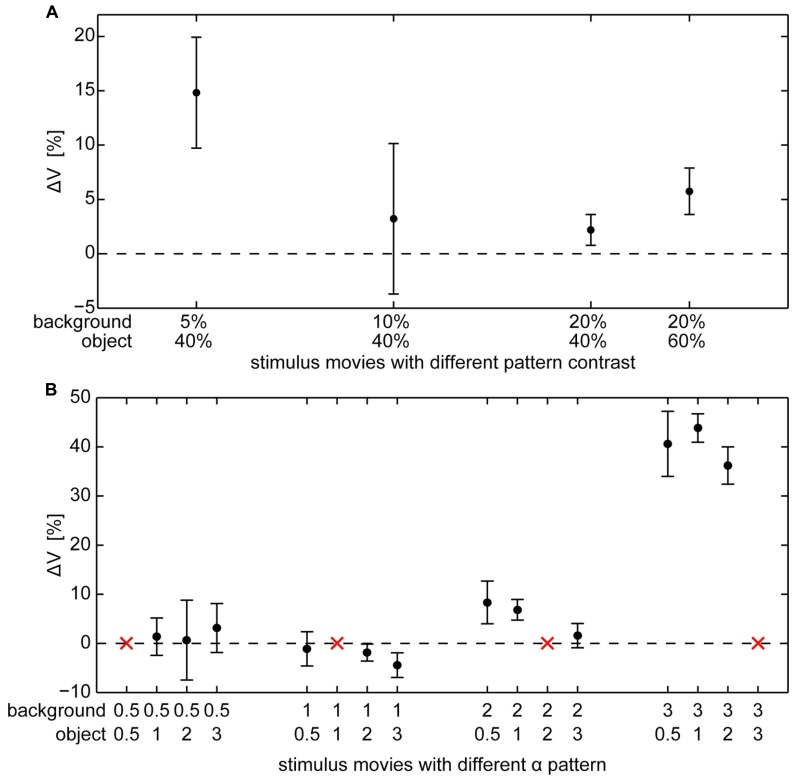
**Object-evoked difference of membrane potential (ΔV) within the “object response intervals” in percent of the response to the characterizing stimulus for individual cells.** Average ΔV (±standard error of the mean) across different cells **(A)** for object and background textures defined by different combinations of contrast (contrasts of object and background are given below the *x*-axis), **(B)** for object and background textures defined by different combinations of spatial frequency (α of object and background texture are given below the *x*-axis).The red “*x*” indicate those combinations where object and background have the same texture and, thus, a zero-response is expected (this condition was not measured to save recording time).

Objects defined exclusively by differences in brightness contrast affected the neural response only if the object texture had a considerably higher contrast than the background texture. For a large contrast difference the object-induced response increments amounted on average to approximately 15% of the maximal response strengths that is elicited by binocular wide-field motion in the HS cell’s preferred direction (**Figure 5A**). We found a relatively large variability of ΔV across different cells. This inter-individual variability covered a similar range as the intra-individual variability across the recordings of single cells. The response variability might be mainly a consequence of inter-individual differences in the sensitivity of HS cells as well as differences due to recording quality. Despite this variability, the mean ΔV values of the different cells (**Table 1**) increased for the combination of a low background contrast (5%) and a high object contrast (40%) as well as a much smaller, however, significant increase in ΔV for the combination of a 20% background contrast and a 60% object contrast (**Figure 5A**). For the other tested combinations of background contrast (10 and 20%) with a 40% object contrast we did not observe a significant object-induced increase in ΔV.

### RESPONSES TO OBJECTS DEFINED BY SPATIAL FREQUENCY CONTENT

Objects defined exclusively by their spatial frequency spectrum led to object-induced response changes only if the object contained more power in the high spatial frequency range than the background. We tested all combinations of our patterns with different spatial frequency content defined by the value α (**Figure 5B**). Again, the inter-individual variability of ΔV was in a similar range as the intra-individual variability of the response. Despite this variability, the mean ΔV values of the different analyzed cells increased considerably for a background texture with a small amount of high spatial frequencies (α = 3) in combination with an object texture with a larger amount of higher spatial frequencies that correspond to lower values of α. Then the object-induced response may increase by more than 40% of the maximal HS responses as induced during binocular wide-field motion in the preferred direction (**Table 1**). This finding indicates that differences in the spatial frequency content between object and background may lead to much larger response increments than even large contrast differences. The combination of a background texture with α = 2 and an object texture with α = 1 led to a much smaller, however, significant increase of ΔV. Under all other conditions no object-induced changes in ΔV could be observed. It should be noted that this result even holds true if the difference in texture between object and background was very large (object α = 3; background α = 0.5, 1, and 2), but of opposite polarity compared to the conditions leading to strong object-induced changes in ΔV. Thus, the polarity of spatial frequency differences between background and object texture seems to be an important determinant of object representation in the cell’s signal.

### INFLUENCE OF BACKGROUND DEPOLARIZATION

As outlined in the previous section, object-induced response changes were only visible if the object texture had more power in the high spatial frequency range than the background texture. This finding indicates that the properties of the background texture may have a higher impact on ΔV than the textural properties of the object. This might be a reasonable expectation, since the background covered much more of the visual field than the object. A possible explanation is that background motion on its own strongly depolarizes the cell and shifts the membrane potential toward its saturation level. Thus, at a high depolarization level of the cell induced by the background an additional object might not be able to depolarize the cell much further. Hence, object-induced effects and, accordingly, the determined ΔV can be expected to be small or even virtually absent.

To scrutinize this hypothesis we took a closer look at the depolarization induced by the motion of the background texture alone. We determined the mean response to the background texture by averaging the cell signal during the object response intervals evoked by the no-object condition stimulus. Since the signal amplitudes differed between different cells, the response values were normalized by dividing them by the response to preferred direction motion of the characterizing stimulus after subtracting the resting potential.

**FIGURE 6:**
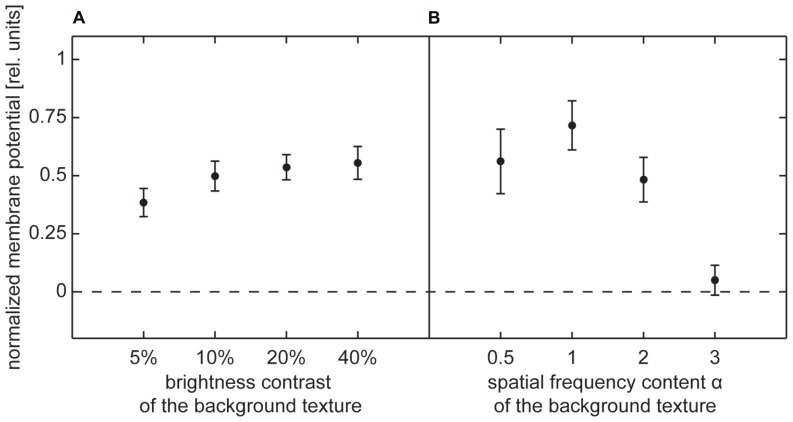
**Depolarization level evoked by background texture (no-object condition) during selected intersaccadic intervals.** Average depolarization (±standard error of the mean) normalized by average depolarization evoked by characterizing stimulus. **(A)** Normalized depolarization induced by contrast background texture, **(B)** normalized depolarization induced by spatial frequency content background texture.

The background response for contrast textures slightly increased with increasing contrast in the low-contrast range, but then approached a constant level (**Figure 6A**). This result is consistent with the well-known dependency of the signal amplitude on brightness contrast (Hausen, 1982b; Egelhaaf and Borst, 1989). The constant response level reached at higher contrasts was not caused by output saturation of the HS cells because it corresponded, on average, to only 50% of the depolarization that could be evoked by the characterizing stimulus during wide-field rotations within large parts of the visual field.

The depolarization level evoked by spatial frequency textures strongly depended on the power of high spatial frequencies. Only very small depolarizations were induced by a background texture containing mainly low frequencies (α = 3). Increasingly higher depolarization levels were obtained for smaller α, i.e., for the textures, in which the frequency content was shifted more toward higher frequencies (**Figure 6B**). However, similar to the results for contrast-defined objects, stimulation with background textures did not reach more than 63% of the depolarization evoked by the characterizing stimulus during wide-field stimulation. Thus, output saturation of the HS cell alone cannot explain why the membrane potential was not much affected by the object, while it was traversing the cell’s receptive field in its preferred direction.

## DISCUSSION

Objects can be discriminated from their background on the basis of a variety of visual cues, such as contrast, color, texture, or relative motion caused by an offset in depth during self-motion. Neurons that are sensitive to these various properties of the environment could combine the response to stimulus changes across this range of different meaningful cues. Thus, neurons that respond to objects in their surroundings might increase their specificity if they were affected by various stimulus cues in parallel (e.g., Nothdurft, 2000). Lobula plate tangential cells (LPTCs), a group of motion sensitive cells of flies, are known to respond to the velocity of wide field motion, but their signal also depends on texture properties like brightness contrast, spatial frequency content and pattern orientation (Hausen, 1982b; Egelhaaf and Borst, 1989; Hausen and Egelhaaf, 1989; Dror et al., 2001; Meyer et al., 2011; O’Carroll et al., 2011). These dependencies are a consequence of the mechanism of elementary motion detection that extracts motion information from the temporal brightness changes as sensed by pairs of neighboring photoreceptors when a pattern is moving across their receptive fields (Reichardt, 1961; Borst and Egelhaaf, 1989; Egelhaaf et al., 1989; Dror et al., 2001).

The texture dependence of the cell signal might be considered as a by-product of motion detection, and, thus, regarded to be detrimental to velocity coding. In contrast to this view, previous studies supported an alternative hypothesis that the texture dependence was a characteristic of motion detection yielding the capacity to code for properties of the environmental structure, especially under the conditions of natural flight dynamics (Kern et al., 2005, 2006; Karmeier et al., 2006; Egelhaaf et al., 2012; Liang et al., 2012). The flight dynamics of flies and several other insect species is shaped by translational flight phases called intersaccadic intervals that contain nearly no rotational motion and last for 30 to 100 milliseconds, both under spatially constrained conditions, but also when the flies could fly straight for few meters or when flying outdoors (Boeddeker et al., 2005). These brief intervals of translational movement alternate with extremely fast saccadic turns of body and head, with amplitudes and directions depending on the environment (Collett and Land, 1975; Schilstra and Van Hateren, 1999; Van Hateren and Schilstra, 1999; Tammero and Dickinson, 2002; Mronz and Lehmann, 2008; Boeddeker et al., 2010; Braun et al., 2010, 2012; Geurten et al., 2010; Kern et al., 2012). Information about the spatial structure of the environment can only be gained from the translational image displacements during the brief intersaccadic intervals. Thus, analyzing how neuronal responses are affected by the presence of objects in the environment during intersaccadic intervals may advance our knowledge on visual information processing in insects. Most previous studies that examined how the response of tangential cells was depending on the textural properties of motion stimuli (Dvorak et al., 1980; Hausen, 1982b) or how they were reacting to temporal changes of the stimulus pattern (Maddess and Laughlin, 1985; Kurtz et al., 2009; Wiederman and O’Carroll, 2011) used basic experimenter-defined stimuli. These differed much from the natural dynamics of the retinal motion patterns generated by the behavior of flies. Only few previous studies analyzed the influence of stimulus discontinuities as induced by an object on the short timescales characteristic of intersaccadic intervals of natural flights (Liang et al., 2008, 2011, 2012). It was shown that objects could increase the intersaccadic responses, if they were defined by an offset in depth and, thus, led to higher retinal velocities than their background.

In our study, we examined for the first time the influence of changes of textural cues that could make an object distinguishable from its background on the short timescale of natural intersaccadic flight phases. We recorded the response of HS cells that are a well-known group of LPTCs, as these neurons strongly respond to motion from front to back which is similar to the retinal image flow during intersaccadic intervals (Hausen, 1982b; Krapp et al., 2001; Kern et al., 2005). We analyzed the effect of objects defined by a single texture property for different combinations of object and background textures. The tested texture properties were brightness contrast and spatial frequency content. These parameters are frequently used to characterize natural images (Ruderman, 1994; van der Schaaf and van Hateren, 1996; Bex and Makous, 2002) and are known to affect the responses of HS cells (Egelhaaf and Borst, 1989; Hausen and Egelhaaf, 1989). The experiments revealed that under the specific dynamic conditions of free flight texture cues of an object were sufficient on their own to alter the intersaccadic responses of HS cells, even if there was no offset in depth and, thus, no discontinuity in the retinal pattern velocity. The response amplitudes induced by differences in the spatial frequency content between object and background were found, at least under certain conditions, to be much larger than those observed for even large contrast differences. This difference is most likely the consequence of the fact that the dependence of the neural response on the spatial frequency content is much more pronounced than that on contrast, and that already a background contrast of 5% depolarizes the cell to a large extent. As a consequence, the neural response changes are strongly affected by the qualitative and quantitative differences in the textures of object and background. In line with previous results (Hausen, 1982b; Egelhaaf and Borst, 1989), higher contrasts and high spatial frequencies lead, within certain ranges, to a larger activation of the cell than low contrasts and spatial frequencies. Therefore, changes of stimulus texture should in principle lead to specific changes of the cell response, irrespective of whether a given texture covers the object or the background. Nonetheless, we found that the texture dependence of object-induced response changes is asymmetric with respect to the parameters of object and background textures: Whereas the combination of background textures with mainly low spatial frequencies and an object texture with high spatial frequencies were causing large object-induced response increments, the inverted combination did not produce noticeable effects, i.e., neither object-induced response decrements nor increments.

The influence of the object texture is strongly related to the overall activation of the cell. Even large changes of texture parameters between object and background do not lead to any further sizable increments in cell activation if the cell is already strongly activated by the background. In contrast, at a lower activation level of the cell objects can induce response increments due to texture changes. These response properties are not a consequence of an output saturation of the HS cell. The activity levels induced by background stimuli of even high contrast and containing high spatial frequencies are just a fraction of the activities that are evoked by our characterizing stimulus, i.e., coherent wide-field rotation in the cell’s preferred direction. This characteristic property of LPTCs can be explained by the concept of gain control (Reichardt et al., 1983; Egelhaaf, 1985; Egelhaaf and Borst, 1989; Borst et al., 1995). Gain control regulates the contribution of the various stimulus parameters to the overall cell response (Grewe et al., 2006). As a result, the neural signal is still able to represent changes in velocity, although the response is not much affected by changes in other stimulus parameters, such as pattern size, contrast or spatial frequency content.

In our study we used object and background textures that were highly artificial and could be varied individually in a targeted way. Nonetheless, both tested pattern parameters are often employed to characterize natural sceneries. The distribution of contrasts has been quantified in numerous analyses of the image statistics of natural scenes (Ruderman, 1994; van der Schaaf and van Hateren, 1996; Bex and Makous, 2002). These studies reveal that low background contrasts that were necessary in our study to allow for object-induced response increments do not frequently occur in natural environments. Background patterns with only relatively low spatial frequencies that were necessary in our experiments for object-induced effects only rarely occur in natural scenes (Ruderman, 1994; van der Schaaf and van Hateren, 1996). However, it should be noticed that natural environments are, in general, much more complex than our stimulus textures. Visual parameters vary much more between different parts of a scene and are combined in a complex manner with the spatial structure in the environment. Thus, changes in contrast might have larger impact in combination with changes of other features of the environment. Furthermore, pattern-dependent fluctuations of the response of fly LPTCs are well-known under constant velocity stimulation with artificial and naturalistic images (Egelhaaf et al., 1989; Single and Borst, 1998; Meyer et al., 2011; O’Carroll et al., 2011; Lindemann and Egelhaaf, 2012). In our study, objects differing from the background in their spatial frequency content did only evoke substantial effects on the overall intersaccadic response on the condition that the background texture was set to parameter values beyond typical natural ranges. In a parameter range frequently occurring under natural conditions the random textures that we used in our experiments did not lead to object induced responses. However, pattern-dependent response modulations are evoked by such natural images (Meyer et al., 2011; O’Carroll et al., 2011). Hence, our results suggest that neither the contrast nor the spatial frequency content of objects and background themselves are sufficient to predict whether object-induced changes of the cell response are evoked. The phase relation of the different spatial frequency components and, thus, the specific spatial arrangement of environmental features are rather likely to play a major role in shaping the cellular responses. The mean response of elementary motion detectors should, in principle, depend only on the frequency content, but not on the phase of the spatial frequency components of stimulus patterns (Borst and Egelhaaf, 1989, 1993; Egelhaaf et al., 1989). This would be the case if the output of elementary motion detectors was pooled across the entire horizontal extent of the visual field. In contrast, the receptive field size of HS cells is limited. Thus, they smooth out the phase information to some degree, but they do not eliminate the influences of the phase relations of the different spatial frequencies completely. Pattern-dependent modulations of the cell response are documented, and they presumably depend on the spatial arrangement of the specific pattern features (O’Carroll et al., 2012). The phase relation of the spatial frequency components of a pattern is responsible for much important information about image features, such as edges. Hence, it might be not surprising that in addition to the spatial frequency content phase information needs to be taken into account for understanding the texture dependency of neural signals.

In conclusion, our study reveals that during the relatively short intersaccadic intervals of natural flights of blowflies objects defined by textural features change the response of HS cells for certain constellations of object and background texture. These are partly related to the depolarization level the background texture evokes during forward flight. Thus, it is not the difference between textures alone, but also the assignment of the specific textures to object or background that are relevant in shaping the object-induced responses. On the whole, these results support the hypothesis that the texture dependence of LPTC responses plays a role in providing the animal with information about the structure of the environment. How this environmental information is processed by downstream mechanisms is not understood so far and requires further examination. It should be noted that the intersaccadic response level of an individual LPTC on its own does not provide unambiguous environmental information as it depends on a variety of stimulus features, such as velocity, contrast and spatial frequency content. The problem of response ambiguity is, however, a general problem of all visual interneurons throughout the animal kingdom that are not affected by just one stimulus feature alone. The ambiguity problem can only be solved by decoding the responses of populations of neurons with somewhat differing stimulus specificities (Zohary, 1992; Pouget et al., 2003; Karmeier et al., 2005). Nevertheless, the representation of texture discontinuities in the neural responses during intersaccadic flight of blowflies emphasizes the significance of these periods for acquiring environmental information.

## Conflict of Interest Statement

The authors declare that the research was conducted in the absence of any commercial or financial relationships that could be construed as a potential conflict of interest.
